# The Agreement Between Blood Pool - Delayed Bone Scintigraphy and Tc-99m Human Immunoglobulin G (HIG) Scintigraphy in the Determination of the Presence and Severity of Inflammatory Arthritis

**DOI:** 10.4274/MIRT.24

**Published:** 2011-08-01

**Authors:** Gulizar Kacar, Cahit Kacar, Firat Gungor

**Affiliations:** 1 Akdeniz University School of Medicine , Department of Nuclear Medicine, Antalya, Turkey; 2 Akdeniz University School of Medicine, Department of Physical Medicine and Rehabilitation, Antalya, Turkey

**Keywords:** Rheumatoid Arthritis, Reactive Arthritis, Technetium 99m HIG

## Abstract

**Objective:** In this study, it was aimed to investigate the agreement between early phase of bone scintigraphy – human immunoglobulin scintigraphy (EPBS-HIG) and late phase bone scintigraphy – HIG (LPBS-HIG) in the determination of the presence and also the severity of inflammatory arthritis.

**Material and Methods: ** Twenty-eight patients (23 female, 5 male; between 19 to 80 years of age) with inflammatory arthritis were included in the study. Tc-99m HIG and blood pool/late phase bone scintigraphies were performed in all patients. In scintigraphic examinations, the joints were scored with the degree of accumulation of the radiopharmaceutical by the semiquantitative analysis (0=Background activity, 1=Faint uptake, 2=Moderate uptake, 3=Marked uptake) which is called as visually active joint score as severity index of the disease. To estimate the agreement between EPBS – HIG and LPBS - HIG in the determination of the presence and severity of inflammatory arthritis, 2x2 kappa coefficients were calculated.

**Results:** Our results showed good agreement between EPBS - HIG for the presence of inflammation (kappa: 0.72) but not for the severity of the disease (kappa: 0.29), poor agreement between LPBS - HIG for both the presence (kappa: 0.51) and severity (kappa: 0.01) of inflammatory arthritis.

**Conclusion:** The blood pool scintigraphy could be used in the investigation of the presence of inflammatory arthritis because the good agreement with HIG and the lower cost but not for the severity of the disease

**Conflict of interest:**None declared.

## INTRODUCTION

Radiopharmaceuticals have been used for detection of inflammation and to evaluate the activity of the arthritis in several arthritic conditions (1,2,3,4,5). Some radiopharmaceuticals accumulate nonspecifically in arthritic joints because of the increased vascular permeability at the site of inflammation or particular physicochemical properties (6). In inflammatory joint disease, the uptake of diphosphonates in bone is either secondary to increased blood flow to periarticular bone, or is related to new bone formation with diphosphonate absorbed on the surface of hydroxyapatite crystals or is a combination of both factors (1). Bone scintigraphy appeared to be a sensitive method for detecting inflammatory joint disease, however the disadvantage of bone scintigraphy is its’ low specificity (1,7,8,9). Other radiopharmaceuticals such as Ga-67 (10,11), radiolabelled leucocytes (12), In-111 chloride (13), ^99m^Tc labelled liposomes (14) were demonstrated to accumulate in inflammed area in arthritis, however these agents have not been used in routine clinical practice (15). Some radiopharmaceuticals were considered as specific targeting agents for inflammation in especially rheumatoid arthritis (RA). Promising results have been reported with radiolabelled CD4, E-selectin antibodies and somatostatin receptor imaging (6). Recently, FDG PET has been used for monitoring response to treatment in RA (16).

HIG scintigraphy has been suggested as a reliable and objective imaging method of joint inflammation. The mechanism of HIG accumulation at the site of inflammation has still to be conclusively determined (1,6). The following hypotheses have been proposed; increased vascular permeability (17), specific trapping of IgG by receptors for immunoglobulins located on inflammatory cells (18), binding of to extracellular matrix proteins (19) and bacterial affinity (20). 

Bone scintigraphy is easy to use and cheaper compare to HIG scintigraphy in routine use. To date, late phase bone scintigraphy (LPBS) has been compared to HIG scintigraphy in inflammatory arthritis. However, there is not enough information about the agreement between early phase of bone scintigraphy (EPBS) and HIG scintigraphy in inflammatory arthritis. In this study, we aimed to investigate the agreement between EPBS-HIG and LPBS-HIG scintigraphy in the determination of the presence and also the severity of inflamatory arthritis. 

## MATERIALS AND METHODS

**Subjects**

The study involved 28 patients (23 female, 5 male; age between 19 to 80 years) with RA (19 patients) diagnosed according to 1987 American College of Rheumatology (ACR) criteria (21) and reactive arthritis (ReA) (9 patients). The range of disease duration was 6 month-35 years and 1 month-1 year in the patients with RA and ReA, respectively. Clinical assessment of arthritis activity was performed with tenderness and swollen of joints. 

**Scintigraphy**

HIG (Mallinckrodt Diagnostica, Holland) was radiolabelled by Tc 99m according to the instructions. Imaging was performed after 4 hours after the iv injection of 555 MBq of the tracer. Whole body scans and anterior spot views of the shoulders, elbows, hands and wrists, hips, knees, ankles and forefeet were acquired at preset times of 5 minutes. 

Bone scans were performed by iv injection of 555 MBq Tc 99m Medronate two days after HIG scintigraphy. Blood pool and late phase static images were acquired at preset times of 2 and 5 minutes, respectively, at the same areas with HIG scintigraphy. Total blood pool imaging time for each patient was between 10 to 12 minutes.

Toshiba GCA 602 A gamma camera equipped with low energy all purpose collimator was used for all acquisitions.

**Scintigraphic Evaluation **

**Figure 1:**
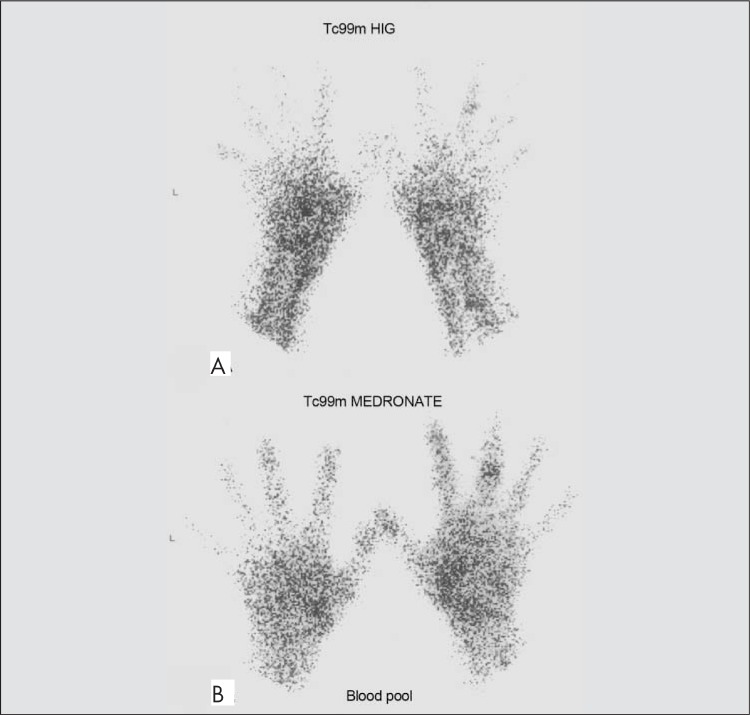
Tc 99m HIG scintigraphy (A) and Tc 99m Medronate bloodpool scintigraphy (B) in the same patientFaint uptake (VAJS= 1) in the proximal interphalangeal joint (at 3^rd^ phalanx of theright hand) and normal uptake of radiopharmaceutical at the level of backgroundactivity (VAJS=0) for other joints of both hands and wrists in HIG and Medronateblood pool scintigraphy

**Figure 2:**
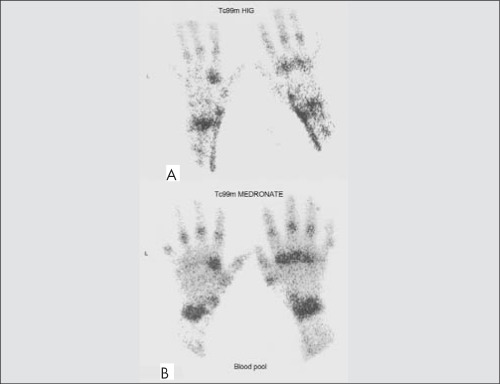
Tc 99m HIG scintigraphy (A) and Tc 99m Medronate bloodpool scintigraphy (B) in the same patientModerate uptake (VAJS=2) in both wrists and metacarpophalangeal joints. Faintand moderate uptake in proximal interphalangeal joints by HIG and Medronateblood pool scintigraphy

**Figure 3:**
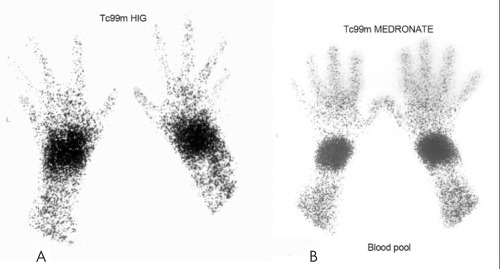
Tc 99m HIG scintigraphy (A) and Tc 99m Medronate bloodpool scintigraphy (B) in the same patientMarked uptake (VAJS=3) in both wrists. Normal uptake in other joints of bothhands by HIG and Medronate blood pool scintigraphy

The scintigrams were evaluated by the consensus of two experienced Nuclear Medicine physicians (GK, FG) who were unaware of the patient’s clinical status. In scintigraphic examinations, the joints were scored with the degree of accumulation of the radiopharmaceutical by the semiquantitative analysis called “visually active joint score” representing the severity index of the disease as follows: 0=Background activity (Figure 1), 1=Faint uptake (mild inflammation) (Figure 1), 2=Moderate uptake (moderate inflammation) (Figure 2), 3=Marked uptake (severe inflammation) (Figure 3). 

Forty-four and 46 joints were investigated for each patient in RA and ReA, respectively by HIG, EPBS, and LPBS. These joints were as follows: shoulders (2), elbows (2), wrists (2), metacarpophalangeal (10), proximal interphalangeal (10), distal interphalangeal (8), hips (2), knees (2), ankles (2), metatarsophalangeal (2) and forefeet (2). Since sacroiliac joint is not a commonly involved joint, it was not evaluated in RA. It was also not possible to score the different metatarsophalangeal and interphalangeal joints of the feet separately, so these joints were taken as a single articular segment. 

Before including the study, detailed information was given to all patients and all patients gave informed consent for the study.

**Statistical Analysis**

To estimate the agreement between HIG – EPBS and HIG – LPBS in the determination of the presence and severity of inflamatory arthritis, 2x2 kappa coefficients were calculated (22). 

## RESULTS

Clinical characteristics and the sum of VAJS for each patient for HIG, EPBS, and LPBS are shown in Table 1. Even the total number of joints evaluated for all patient was 1250 (44 joints x 19 patients with RA; 46 joints x 9 patients with ReA), it was not possible to compare HIG–EPBS and HIG–LPBS with each other for all joints due to the variations in acquisition and technical problems. The number of joints compared with each other in HIG–EPBS and HIG–LPBS were 415 and 473, respectively. 

Tables 2,3,4,5 show the agreement results between HIG - EPBS and HIG - LPBS in the determination of the presence and severity of arthritis.

## DISCUSSION

The availability of an objective and reproducible method to evaluate the activity of the arthritis would be of great value in management of patients and assessment of therapeutic effects (6). Many of the studies have shown that nonspecific polyclonal human IgG scintigraphy is a useful test for localizing and detecting inflammatory joint activity and inflammation (23,24,25,26,27,28,29,30). 

In the literature, LPBS has been compared to HIG scintigraphy in inflammatory arthritis for only in the determination of the presence of inflammatory arthritis (24,29). However, there is not enough information about the agreement between LPBS - HIG scintigraphy in the determination of the severity of disease. In addition, in the literature, the agreement between EPBS – HIG for the determination of the presence and also the severity of the disease are not well known, either. The present study has adressed to clarify this point. Our study showed that there was a good agreement between HIG and EPBS in the determination of the presence of arthritis (percent of overall agreement: 0.86, kappa: 0.72). This means that HIG and EPBS are in agreement in 356 of 415 joints. However, the agreement of HIG and EPBS was poor in the evaluation of the severity of arthritis (percent of overall agreement: 0.47, kappa: 0.29). In another words, only 256 of 415 joints showed good agreement in HIG and EPBS for the determination of the severity of arthritis. Klett et al (30) also showed excellent agreement between HIG and EPBS in the determination of the presence of arthritis but they did not investigate the agreement for the severity of arthritis. In the present study, poor agreement was found between HIG - LPBS in the determination of the presence (kappa: 0.51) and severity (kappa: 0.01) of inflammatory arthritis. In the literature, there is not any information about the agreement of two modalities for the severity of arthritis. Many studies also indicated that HIG scintigraphy, when compared to bone scintigraphy, is a more specific method to detect synovitis and shows differentiation between different degrees of arthritis acitivity (2,15,24). HIG and MDP are completely different radiopharmaceuticals in terms of uptake mechanisms. Bois et al suggest that two scintigraphic techniques reflect different processes. HIG scintigraphy measures inflammation and bone scintigraphy measures bone turnover. The differences between the results of the two scintigraphic techniques can be explained by the different uptake mechanisms of the radiopharmaceuticals (15). While increased vascular permeability (17), specific trapping of IgG by receptors for immunoglobulins located on inflammatory cells (18), binding of to extracellular matrix proteins (19) are factors proposed for HIG, only increased vascular permeability and metabolic activity of bone are responsible for EPBS and LPBS, respectively, in inflammatory arthritis (1). Our results showed that the agreement between HIG – EPBS is better than HIG – LPBS in terms of the determination of the presence and severity of the disease. In the LPBS, the main affected factor of accumulation of tracer is the bony turnover (15). Even after improvement of synovitis, since neighboring bony turnover would still continue, LPBS would show increased tracer accumulation. 

The main drawback of the present study is absence of a gold standart test for investigation of synovitis of arthtritic joints. In the present study, patients were diagnosed according to ACR criteria for RA, and clinical-laboratory data for ReA. The histologic examination is the gold standart modality for evaluating synovitis. Because of its invasiveness, there is only one study comparing the results of HIG scintigraphy with histologic examination (23). In this study, false positive as well as false negative results are shown with a higher number of false positive results. However, the present study had addressed to investigate only the agreement between two imaging modalities for the determination of the presence and the severity of the inflamatory arthritis rather than the primary diagnosis. 

## CONCLUSION

Our results showed good agreement between HIG – EPBS for the presence of inflammation but not for the severity of the disease and poor agreement between HIG – LPBS for both the presence and severity of inflammatory arthritis. The blood pool scintigraphy could be used for the investigation of the presence of inflammatory arthritis because of the good agreement with HIG and lower cost but not for the severity of the disease. 

## Figures and Tables

**Table 1:**
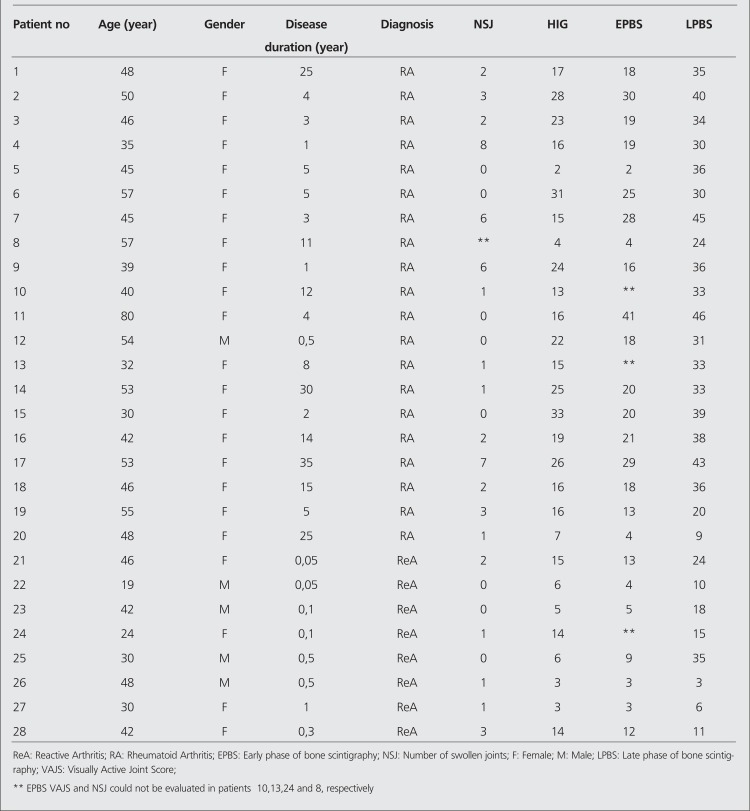
Clinical characteristics and the sum of visually active joint scores for HIG, EPBS, and LPBS of each patient

**Table 2:**
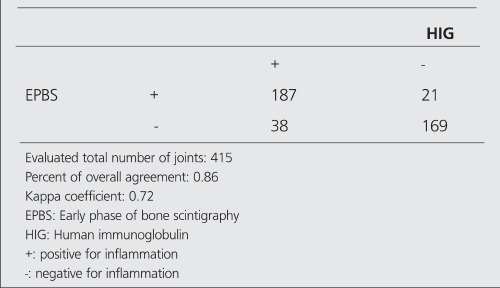
The number of positive and negative joints in HIG and EPBS

**Table 3:**
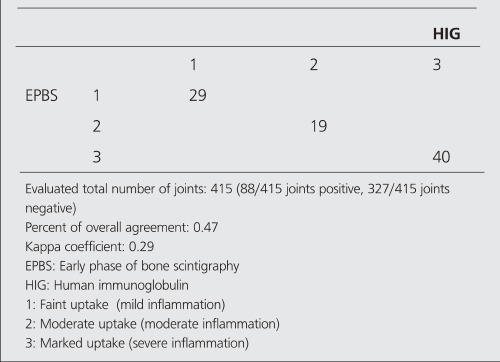
The number of joints (showing positive uptake) in terms ofseverity of arthritis in HIG and EPBS

**Table 4:**
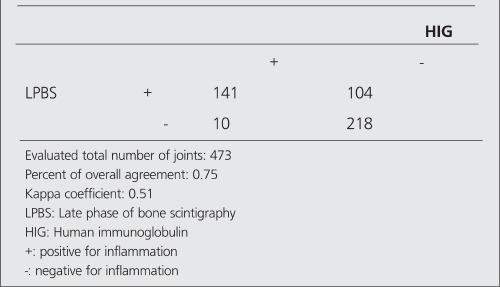
The number of positive and negative joints in HIG and LPBS

**Table 5:**
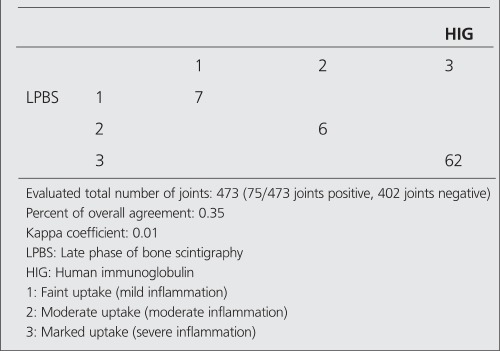
The number of joints (showing positive uptake) in terms ofseverity of arthritis in HIG and LPBS
